# The Association between Ideal Cardiovascular Health Metrics and Extracranial Carotid Artery Stenosis in a Northern Chinese Population: A Cross-Sectional Study

**DOI:** 10.1038/srep31720

**Published:** 2016-08-30

**Authors:** Zhiru Hao, Yong Zhang, Yongming Li, Jinbo Zhao, Yong Zhou, Jing Qiu, Ruiping Zhao, Jiang Hu

**Affiliations:** 1 Department of Cardiology, Central Hospital of Baotou, Baotou, 014040 China; 2 Graduate school, Baotou Medical College, Baotou, 014040 China; 3 Department of Neurology, Beijing Tiantan Hospital, Capital Medical University, Beijing, 100050 China; 4 School of Public Health, Ningxia Medical University, Yinchuan, 750004 China; 5 Department of Surgery, Central Hospital of Baotou, Baotou, 014040 China

## Abstract

Past epidemiologic studies have indicated that the ideal cardiovascular health (CVH) metrics are associated with a lower risk of cardiovascular diseases (CVDs) and stroke. Carotid artery stenosis (CAS) causes approximately 10% of ischemic strokes. The association between ideal CVH and extracranial CAS has not yet been assessed. In the current study, extracranial CAS was assessed by carotid duplex ultrasonography. Logistic regression was used to analyze the association between ideal CVH metrics and extracranial CAS. A total of 3297 participants (52.2% women) aged 40 years and older were selected from the Jidong community in China. After adjusting for sex, age and other potential confounds, the odds ratios (95% confidence intervals) for extracranial CAS were 0.57 (0.39–0.84), 0.46 (0.26–0.80) and 0.29 (0.15–0.54), and for those quartiles, quartile 2 (9–10), quartile 3 (11) and quartile 4 (12–14), respectively, compared with quartile 1 (≤8). This negative correlation was particularly evident in women and the elderly (≥60 years). This cross-sectional study showed a negative correlation between the ideal CVH metrics and the prevalence of extracranial CAS in northern Chinese adults.

Carotid artery stenosis (CAS) causes approximately 10% of ischemic strokes^1^. Approximately 7% of first ischemic strokes were associated with extracranial CAS^2^. Data from the Global Burden of Disease Study 2010 (GBD2010) indicated that the leading cause of death in China is stroke^3^. Furthermore, previous studies have indicated that many patients with CAS have a greater risk of dying due to myocardial infarction (MI) than from stroke^4^,^5^. In the Framingham Heart Study, the prevalence of CAS in people aged 66 to 93 years was 7% in women and 9% in men^6^. CAS refers to an atherosclerotic narrowing specifically of the extracranial CAS, the internal CAS or the common CAS^7^. Genetic susceptibility may play a key role in the development of CAS, and there may be different pathophysiologies for the different CAS locations^8^,^9^. However, there are other possible factors in the development of CAS, including differences in the prevalence of risk factors or of particular life styles across different ethnic populations^10^. Due to the high prevalence of extracranial CAS, the study of risk factors and protective measures for extracranial CAS has particular public health significance in China.

The American Heart Association (AHA) has defined ideal cardiovascular health (CVH) as including four ideal health behaviors (body mass index, physical activity, healthy diet, and smoking status) and three ideal health factors (total cholesterol, blood pressure and fasting blood glucose)^11^. Worldwide, cardiovascular diseases (CVDs) remain a major public health burden and are expected to increase over the next 10 years as the population ages^11^,^12^. Several studies have suggested that the ideal CVH metrics were remarkably negatively associated with the total incidence of CVD and stroke^11^,^13^,^14^,^15^. In addition, a study based on 5440 Chinese adults found a clear gradated negative relationship between a higher number of ideal CVH metrics and a lower prevalence of asymptomatic intracranial carotid artery stenosis (ICAS)^16^. However, the association between the ideal CVH metrics in the AHA definition and extracranial CAS is still unclear. Therefore, a cross-sectional analysis was conducted to explore this association in a northern Chinese cohort that consisted of 3297 adult participants.

## Results

From the initial sample of 4428 participants, 369 participants were excluded because of physical disabilities or because they did not provide informed consents and 762 participants were excluded because of incomplete data on their health factors, health behaviors or other variables. Finally, 3297 participants were included in the final analysis.

Table 1 shows the basic characteristics of the participants stratified by sex.

Women tended to be younger, lower educated and of average income. Participants with heavy alcohol consumption were almost always male (99% vs 1%). In addition, parameters such as BMI, SBP, DBP, FPG and TG were higher in men, while TC was lower in men, compared to the values of those parameters in women.

Table 2 shows the association between each CVH metric and the prevalence of extracranial CAS.

After adjusting for sex, age, alcohol use, average monthly income of each family member, education level and the other six cardiovascular health metrics, we found that the ideal smoking status, blood pressure, total cholesterol and fasting blood glucose metrics were significantly associated with a lower prevalence of extracranial CAS (OR = 0.55, 95% CI: 0.35–0.85, *P* = 0.007; OR = 0.46, 95% CI: 0.25–0.84, *P* = 0.012; OR = 0.45, 95% CI: 0.24–0.85, *P* = 0.014; OR = 0.55, 95% CI: 0.34–0.90, *P* = 0.017; respectively). In further stratified analyses, the negative associations of extracranial CAS with the ideal blood pressure and ideal total cholesterol were significant (*P* ≤ 0.05) in men but not in women, and the negative correlation of extracranial CAS with the ideal total cholesterol and ideal fasting blood glucose were only found in middle-aged groups (40–60 years of age). Similarly, the negative correlation with the ideal smoking status was particularly evident in women and the elderly (≥60 years).

Table 3 shows the relationship between the total score of the ideal CVH metrics and the prevalence of extracranial CAS.

After adjusting for sex, age, alcohol use, average monthly income of each family member, and education level, participants in the highest quartile of the ideal CVH metrics summary score had a lower prevalence of extracranial CAS compared to those in the lowest quartile of the summary score (OR = 0.29, 95% CI: 0.15–0.54, *P* < 0.001). The stratified analyses found that this significant negative correlation was still evident particularly in men (*P* < 0.001) and the elderly (≥60 years) (*P* < 0.001).

## Discussion

The main strengths of the present study are as follows. 1) The preliminary analysis showed that the ideal smoking status, blood pressure, total cholesterol and fasting blood glucose metrics were significantly associated with a low prevalence of extracranial CAS in the total population compared to the poor group. 2) Our data indicated that individuals in the highest quartile of the total ideal CVH metrics had a 71% reduced risk of developing extracranial CAS compared to those in the lowest quartile, after adjusting for potential confounds (sex, age, alcohol use, average monthly income of each family member and education level). 3) A similar negative correlation was observed in subgroups of women and the elder (≥60 years). ECAS is a subclinical indicator for stroke and cerebrovascular diseases. The association between ECAS and CVD suggests that CVD may primarily contribute to ECAS and then lead to cerebrovascular diseases via subclinical cerebrovascular changes. To our knowledge, this study is novel in its exploration of the potential association between the ideal CVH and extracranial CAS in an adult Chinese population.

Previous studies have investigated the combined association of extracranial CAS prevalence and different risk factors on atherosclerosis. The Framingham Heart Study, a multivariate logistic regression model, showed that age, cigarette smoking, systolic blood pressure, and cholesterol were independently related to carotid atherosclerosis^6^,^17^. Similar to previous studies^17^,^18^,^19^,^20^, we showed that the ideal metric of smoking status was inversely associated with extracranial CAS. In the Cardiovascular Health Study^19^, the severity of CAS was greater in current smokers than in former smokers, and the severity of CAS was significantly associated with pack-years of exposure to tobacco. Similarly, the Framingham Heart Study found that extracranial CAS was correlated with the quantity of cigarettes smoked over time, especially in women^17^, which is consistent with our findings. Although the association in our study did not reach statistical significance (*P* = 0.086) in men, there was a highly significant trend across the total participants (*P* = 0.007). There was an OR of 1.33 (95% CI: 0.37–4.77) for intermediate smoking compared to sparse smoking that could have been caused by the small sample size of smokers, which could have led to insufficient statistical power.

According to our study, the ideal metrics of blood pressure, total cholesterol and fasting blood glucose had inverse relationships with extracranial CAS. Previous studies on risk factors for extracranial CAS also showed similar results^17^,^21^,^22^,^23^. The Framingham Heart Study indicated that there was a 2-fold increased risk of carotid stenosis for every 20 mmHg increase in SBP^17^. Sutton-Tyrrell *et al*. found that a SBP of ≥160 mmHg was the strongest independent predictor of extracranial CAS, especially in the elderly^21^. Our study confirmed this association, in men but not in women, a finding that can be explained by physique differences between the sexes. The MESA study^24^ showed that total cholesterol was strongly associated with a carotid plaque lipid core and the Framingham Heart Study^17^ found that the relative risk (RR) of CAS was >25%, and increased approximately 1.1 for every 10 mg/dL increase in total cholesterol, which are findings that are both consistent with our results, particularly among middle-aged adults (40–60 years of age) compared to the elderly. Cholesterol levels often decline in the elderly, which may be a cause of the lower association in our study. In addition, our finding of a negative association between the ideal FPG and extracranial CAS is supported by results from the IRAS (Insulin Resistance Atherosclerosis Study)^25^, suggesting that the progression of carotid atherosclerosis is accelerated in persons with type 2 diabetes and that this increased rate of atherosclerosis is partially explained by the atherogenic risk factor profile associated with diabetes. Similarly, the Cardiovascular Health Study^23^ also reported that diabetes was associated with carotid IMT and the severity of CAS. In the present study, significant negative correlations between the ideal CVH body mass index, physical activity or health diet metrics and extracranial CAS were not observed. The previous study also found that physical activity was not associated with carotid atherosclerosis^26^.

Previous studies found inverse associations between improving diet and weight management and the development of carotid atherosclerosis^27^,^28^,^29^, which was in contrast to our study. The present study indicated that there was no significant relationship between diet and CAS (OR 0.81, 95% CI: 0.46–1.43; in those with a poor diet score compared to those with an ideal diet score), which is contrary to a previous study (ref. 27) in regard to the OR value. This inconsistency may be explained by the following two reasons. First, the two studies used different definitions of ideal diets, and in particular, the different ranges led to different cut-offs for ideal, intermediate and poor diet scores. Second, the association between the ideal diet score and CAS may be smaller in a Chinese population than in other populations, and therefore, our study may not have enough statistical power to detect the association (OR 0.81, 95% CI: 0.46–1.43, *P* = 0.28). In our study, participants were Han-ethnic Chinese, who probably have different physiques and lifestyles than other ethnic populations.

Our study indicated a significant negative correlation between the ideal CVH metrics and the prevalence of extracranial CAS. Extracranial CAS has several causes including atherosclerosis, fibromuscular dysplasia (FMD), cystic medial necrosis, arteritis, and dissection, with the most frequent cause being atherosclerosis^29^. Atherosclerosis is a systemic disease and patients with extracranial CAS typically have an escalated risk of other adverse cardiovascular events, including myocardial infarction (MI), peripheral arterial disease (PAD), and death^30^,^31^,^32^. Risk factors associated with extracranial CAS, such as cigarette smoking, hypertension, diabetes and hypercholesterolemia, are the same as those for atherosclerosis located elsewhere, but differences exist in the relative contribution to the risk in the various vascular beds. Therefore, it makes sense that maintaining the ideal CVH metrics may be the most effective means to avoid extracranial CAS. This is the idea of primordial prevention^11^.

There are potential limitations of our study. First, the Jidong community involves mainly regional populations, so the results may not be generalizable across the nation due to geographic variations, and different educational, economic and cultural backgrounds. Second, this study was a cross-sectional study that did not have the capacity to evaluate causal relationships between the ideal CVH metrics and extracranial CAS. Therefore, our study can only speculate that maintaining CVH may be beneficial in reducing the risk of atherosclerosis. Prospective studies are needed to further examine the causality links. Third, carotid duplex ultrasonography is a noninvasive vascular test that may underestimate the severity of stenosis (especially in cases of less than 50% stenosis)^33^. However, carotid duplex ultrasonography is widely used for initial evaluations and to estimate CAS, and experienced sonographers are able to provide accurate and relatively inexpensive assessment of CAS via this method^34^. Fourth, the cardiovascular behavior measures, such as physical activity, smoking, and dietary intake, were from self-reported questionnaires, so misclassification was possible, especially in regard to diet and physical activity. Finally, extracranial CAS involves complex mechanisms like different etiologies and pathogeneses, which our study did not identify. Therefore, different potential risk factors and preventive strategies for CAS need to be explored further.

In conclusion, the ideal CVH metrics were significantly associated with the prevalence of extracranial CAS in northern Chinese adults, especially in women and the elderly (≥60 years). This negative correlation indicates that maintaining an ideal CVH may be of great value in preventing extracranial CAS. Prospective studies are needed to further investigate these questions.

## Method

### Study Design and Participants

From July 2013 to August 2014, 9,078 participants (man 4,768, aged 18–82 years old) were enrolled in the study cohort and comprised employees (including those who were retired) and their family members from the Jidong Co. Ltd, a large Oilfield in Hebei Province, China. In our study, the inclusion criteria were: (1) aged 40 years or older; and (2) without stroke, transient ischemic attack, and myocardial infarction (MI). Among the 9078 potential participants, 4428 satisfied the above inclusion criteria. In addition, participants were excluded from this study according to following exclusion criteria: (1) incomplete data on health factors or behaviors, or not providing informed consent, and (2) physical disabilities.

### Ethics Statement

The study was performed according to the guidelines of the Helsinki Declaration, with the approval of the Ethics Committee of the Jidong Oilfield Hospital. All participants agreed to study participation and provided informed consents.

### Assessment of Cardiovascular Health Metrics

Information about smoking, physical activity, and dietary intake was collected via questionnaires. Dietary habits were assessed with a brief semi-quantitative food frequency questionnaire^35^,^36^, which had definitions similar to the AHA definitions of dietary habits (see Table 4). The definition of an ideal diet score in this study was as reported in our previous publication^37^. According to the AHA definitions^11^, we further categorized smoking status, physical activity and dietary intake into three groups: “ideal”, “intermediate” or “poor”. Smoking was classified as ideal (never or quit smoking >12 months previously), intermediate (quit smoking ≤12 months ago), or poor (currently smoking); physical activity was classified as ideal (≥150 min/week of moderate intensity or ≥75 min/week of vigorous intensity), intermediate (1–149 min/week of moderate intensity or 1–74 min/week of vigorous intensity), or poor (none). Healthy diet behaviors were classified as ideal (4–5 components), intermediate (2–3 components) or poor (0–1 component).

Body mass index (BMI) was calculated based on the weight (accurate to 0.1 kg) and height (accurate to 0.1 cm) measurements of the participants, as body weight (kg)/the square of height (m^2^). Participants’ blood pressure was measured twice by experienced research nurses following 5 minutes of rest in a seated position using a mercury sphygmomanometer with a cuff of the appropriate size. The averages of the two systolic blood pressure (SBP) and diastolic blood pressure (DBP) readings were used for the analysis. If the deviation of the two measurements was more than 5 mm Hg, an additional reading was taken and the average of the three readings was used. All blood pressure was measured using the right arm. Hypertension was defined as the presence of a history of hypertension, using antihypertensive medication, a SBP ≥ 140 mm Hg, or a DBP ≥ 90 mm Hg. According to the AHA definitions^11^, BMI was classified as ideal (<25 kg/m2), intermediate (25 to 29.9 kg/m2) or poor (≥30 kg/m2); blood pressure was classified as ideal (SBP < 120 mmHg and DBP < 80 mmHg and untreated), intermediate (120 mm Hg ≤ SBP ≤ 139 mmHg, 80 mmHg ≤ DBP ≤ 89 mmHg, or treated to SBP/DBP < 120/80 mmHg), or poor (SBP ≥ 140 mmHg, DBP ≥ 90 mmHg, or treated to SBP/DBP > 120/80 mmHg).

Blood samples were collected from the antecubital vein by trained research nurses in the morning following overnight fasting and were transfused into vacuum tubes containing EDTA. The tubes were then centrifuged for 10 minutes at 3000 rotations per minute at 25 °C. After separation, plasma samples were used within 4 hours. All biochemical variables, including total cholesterol and fasting blood glucose, were measured using an autoanalyzer (Olympus, AU400, Japan) in the central laboratory of the Jidong Oilfield Hospital. According to the AHA definitions^11^, fasting blood glucose was classified as ideal (<100 mg/dL and untreated), intermediate (100 to 125 mg/dL or treated to <100 mg/dL), or poor (≥126 mg/dL or treated to ≥100 mg/dL); total cholesterol was classified as ideal (<200 mg/dL and untreated), intermediate (200 to 239 mg/dL or treated to <200 mg/dL), or poor (≥240 mg/dL or treated to ≥200 mg/dL).

### Assessment of Extracranial Carotid Artery Stenosis

All participants (≥40 years) underwent carotid duplex ultrasonography in a supine position, with their head turned to the contralateral side. All carotid scans were performed by two independent sonographers with ultrasounds. The sonographers were blind to the baseline information of the participants. Carotid duplex ultrasound modalities combined 2-dimensional real-time imaging with Doppler flow analysis to evaluate the vessels of interest (typically the cervical portions of the common and internal carotid arteries) and to measure blood flow velocity. Extracranial CAS was defined by a peak systolic blood flow velocity ≥125 cm/s and a vertical artery peak systolic blood flow velocity of ≥170 cm/s in the common carotid artery or internal carotid artery. Extracranial CAS was graded according to the diagnostic criteria identified by the Society of Radiologists in the Ultrasound Consensus Conference in 2003. In our study, the degree of CAS was classified as normal (no stenosis) or stenosis (<50% stenosis, ≥50% stenosis or occlusion) involving at least one internal or common CAS.

### Assessment of Potential Covariates

Biographical information (age, race, sex, education level, average monthly income of each family member, alcohol use and disease history) was collected via questionnaires at the baseline visit. Participants were divided into two groups according to age: 40–59 years and ≥60 years. Participants’ education level was categorized as “illiterate or primary”, “middle/high school” or “college graduate or above”. The average monthly income of each family member was reported to be “ ≤¥3,000”, “¥3,001–5,000” or “ ≥¥5,001”. Heavy alcohol consumption was defined as a daily intake of at least 100 ml of liquor (equivalent to 240 ml of wine or 720 ml of beer) for more than a year. The existence of a history of stroke or MI was defined as any self-reported previous physician’s diagnosis of stroke or MI.

### Statistical Analyses

Statistical analyses were performed using SAS software, version 9.4 (SAS Institute, Cary, North Carolina, USA). Categorical variables were described using percentages and compared with Chi square tests. Continuous variables were described using means (standard deviation [SD]) and compared with ANOVAs. Logistic regression was used to estimate the prevalence of extracranial CAS across the subgroups of each ideal CVH metric by calculating the odds ratio (OR) and 95% confidence interval (CI). Adjustments were made for five variables (sex, age, alcohol use, average monthly income of each family member, and education level) that were identified as potential confounds of the risk factors for extracranial CAS^14^,^16^,^38^. In addition, the composite score of the seven CVH metrics was quantified by adding the following numeric values assigned to each component based on category: 0 = poor, 1 = intermediate, and 2 = ideal^39^. The prevalence of extracranial CAS was analyzed using the total score of the CVH metrics inserted into the models as quartiles (with the lowest quartile as the reference), using logistic regression. All statistical tests were 2-sided, and significance levels were 0.05.

## Additional Information

**How to cite this article**: Hao, Z. *et al*. The Association between Ideal Cardiovascular Health Metrics and Extracranial Carotid Artery Stenosis in a Northern Chinese Population: A Cross-Sectional Study. *Sci. Rep*. **6**, 31720; doi: 10.1038/srep31720 (2016).

## Figures and Tables

**Table 1:** Characteristics of Study Participants Stratified by Sex.

	ALL (n = 3297)	Man (n = 1576)	Women (n = 1721)	***P***-value
Income, ¥/month n(%)				<0.001
≤¥3000	1642(49.8)	714(43.5)	928(56.5)	
¥3001–5000	1451(44.0)	746(51.4)	705(48.6)	
≥¥5001	204(6.2)	116(56.9)	88(43.1)	
Education level n(%)				<0.001
Illiteracy/primary	242(7.3)	85(35.1)	157(64.9)	
Middle school	1852(56.2)	823(44.4)	1029(55.6)	
College/University	1203(36.5)	668(55.5)	535(44.5)	
Alcohol use n(%)				<0.001
Yes	103(3.1)	102(99.0)	1(1.0)	
No	3194(96.9)	1474(46.1)	1720(53.9)	
BMI(kg/m^2^)	24.9 ± 3.28	25.6 ± 2.98	24.3 ± 3.41	<0.001
SBP(mmHg)	131 ± 19.8	133 ± 18.81	129 ± 20.5	<0.001
DBP(mmHg)	83.7 ± 12.2	87.8 ± 12.7	79.9 ± 12.6	<0.001
FPG(mg/dl)	5.51 ± 1.37	5.67 ± 1.52	5.37 ± 1.20	<0.001
TC(mg/dl)	4.69 ± 0.89	4.62 ± 0.85	4.76 ± 0.92	<0.001
TG(mg/dl)	1.71 ± 1.42	1.92 ± 1.62	1.52 ± 1.06	<0.001

BMI, body mass index; SBP, systolic blood pressure; DBP, diastolic blood pressure; FPG, fasting blood pressure; TC, total cholesterol; TG, triglyceride.

**Table 2:** Odds Ratio with 95% CI of Each Component of Cardiovascular Health Metric for Extracranial Carotid Artery Stenosis Stratified by Age and Sex.

Metrics	Total	Sex		Age(years)	
		Man	Women	40~59	≥60
Smoking					
Poor	1.00	1.00	1.00	1.00	1.00
Intermediate	1.33(0.37–4.77)	1.66(0.46–6.04)	—*	0.99(0.12–7.99)	1.14(0.21–6.33)
Ideal	0.55(0.35–0.85)	0.67(0.42–1.06)	0.14(0.04–0.45)	0.82(0.45–1.46)	0.45(0.24–0.86)
*P*-value	0.007	0.086	0.001	0.492	0.016
BMI					
Poor	1.00	1.00	1.00	1.00	1.00
Intermediate	0.91(0.48–1.72)	1.20(0.47–3.02)	0.88(0.35–2.25)	0.98(0.41–2.37)	0.90(0.35–2.36)
Ideal	1.03(0.54–1.98)	1.77(0.69–4.51)	0.58(0.21–1.56)	1.18(0.48–2.91)	1.17.(0.44–3.10)
*P*-value	0.926	0.235	0.278	0.721	0.751
Physical activity					
Poor	1.00	1.00	1.00	1.00	1.00
Intermediate	0.59(0.23–1.55)	0.76(0.25–2.31)	0.27(0.03–2.18)	—	1.77(0.58–5.36)
Ideal	1.01(0.69–1.47)	1.21(0.74–1.97)	0.70(0.35–1.30)	1.45(0.86–2.45)	0.77(0.44–1.35)
*P*-value	0.979	0.460	0.258	0.163	0.358
Diet					
Poor	1.00	1.00	1.00	1.00	1.00
Intermediate	1.17(0.72–1.55)	1.21(0.68–2.13)	1.25(0.49–3.18)	1.91(0.58–2.06)	1.25(0.60–2.60)
Ideal	0.81(0.46–1.43)	0.80(0.40–1.62)	0.87(0.31–2.48)	0.73(0.34–1.57)	0.86(0.36–2.05)
*P*-value	0.459	0.542	0.797	0.472	0.734
Total cholesterol					
Poor	1.00	1.00	1.00	1.00	1.00
Intermediate	0.71(0.36–1.37)	0.65(0.27–1.57)	0.69(0.23–2.08)	0.41(0.20–1.11)	1.49(0.48–4.65)
Ideal	0.45(0.24–0.85)	0.29(0.13–0.69)	0.74(0.27–2.07)	0.39(0.18–0.85)	0.72(0.24–2.21)
*P*-value	0.014	0.005	0.570	0.018	0.571
Blood pressure					
Poor	1.00	1.00	1.00	1.00	1.00
Intermediate	0.64(0.44–0.93)	0.63(0.40–1.01)	0.60(0.31–1.19)	0.69(0.40–1.18)	0.61(0.35–1.04)
Ideal	0.46(0.25–0.84)	0.43(0.19–0.96)	0.51(0.19–1.37)	0.48(0.22–1.04)	0.34(0.12–0.97)
*P*-value	0.012	0.040	0.180	0.064	0.043
Fasting blood glucose					
Poor	1.00	1.00	1.00	1.00	1.00
Intermediate	0.62(0.37–1.06)	0.51(0.26–1.04)	0.72(0.29–1.75)	0.41(0.19–0.87)	1.06(0.49–2.28)
Ideal	0.55(0.34–0.90)	0.70(0.38–1.27)	0.34(0.14–0.83)	0.39(0.20–0.76)	0.71(0.34–1.49)
*P*-value	0.017	0.243	0.023	0.005	0.361

CI: confidence interval.

The following potential confounders were adjusted for each OR: sex, age, alcohol use, average monthly income of the family members, education level and the other six cardiovascular health metrics.

^*^None of the women were former smokers but have quit smoking for ≤12 months.

**Table 3:** Associations of Extracranial Carotid Artery Stenosis with Ideal Cardiovascular Health Metrics Stratified by Age and Sex.

Metrics	Prevalence of Extracranial CAS(%)	Total	Sex		Age (years)	
			Man	Women	40~59	≥60
Quartile 1 (≤8)	46.9	1.00	1.00	1.00	1.00	1.00
Quartile 2 (9–10)	33.3	0.57(0.39–0.84)	0.68(0.42–1.09)	0.39(0.19–0.77)	0.67(0.38–1.16)	0.55(0.32–0.94)
Quartile 3 (11)	11.6	0.46(0.26–0.80)	0.40(0.18–0.89)	0.43(0.19–0.99)	0.71(0.35–1.45)	0.31(0.13–0.76)
Quartile 4 (12–14)	8.2	0.29(0.15–0.54)	0.51(0.23–1.12)	0.11(0.04–0.34)	0.38(0.17–0.88)	0.21(0.07–0.60)
*P* for trend		<0.001	0.012	<0.001	0.026	<0.001

The following potential confounders were adjusted for each OR: sex, age, alcohol use, average monthly income of the family members, education level.

**Table 4:** Definition of Ideal Diet Score (>20 Years of Age) by the AHA and the Criteria Used in the Study.

Item	ideal diet score by AHA	ideal diet score in this study
Fruits and vegetables	4.5 cups per day	4.5 or more servings per day
Fish	two 3.5-oz servings per week (preferably oily fish)	2 or more servings per week
Fiber-rich whole grains (1.1 g of fiber per 10 g of carbohydrate)	three 1-oz-equivalent servings per day	3 or more servings per day
Salt	1500 mg per day of Sodium	less than 6 gram per day of salt intake
Sugar	450 kcal (36 oz) per week of sugar-sweetened beverage	sugary drinks once a week or less
